# The impact of heatwaves on emergency department visits in Brisbane, Australia: a time series study

**DOI:** 10.1186/cc13826

**Published:** 2014-04-09

**Authors:** Ghasem Sam Toloo, Weiwei Yu, Peter Aitken, Gerry FitzGerald, Shilu Tong

**Affiliations:** 1 School of Public Health and Social Work, Queensland University of Technology, Victoria Park Road, Kelvin Grove, QLD 4059, Australia

## Abstract

**Introduction:**

The acute health effects of heatwaves in a subtropical climate and their impact on emergency departments (ED) are not well known. The purpose of this study is to examine overt heat-related presentations to EDs associated with heatwaves in Brisbane.

**Methods:**

Data were obtained for the summer seasons (December to February) from 2000–2012. Heatwave events were defined as two or more successive days with daily maximum temperature ≥34°C (HWD1) or ≥37°C (HWD2). Poisson generalised additive model was used to assess the effect of heatwaves on heat-related visits (International Classification of Diseases (ICD) 10 codes T67 and X30; ICD 9 codes 992 and E900.0).

**Results:**

Overall, 628 cases presented for heat-related illnesses. The presentations significantly increased on heatwave days based on HWD1 (relative risk (RR) = 4.9, 95% confidence interval (CI): 3.8, 6.3) and HWD2 (RR = 18.5, 95% CI: 12.0, 28.4). The RRs in different age groups ranged between 3–9.2 (HWD1) and 7.5–37.5 (HWD2). High acuity visits significantly increased based on HWD1 (RR = 4.7, 95% CI: 2.3, 9.6) and HWD2 (RR = 81.7, 95% CI: 21.5, 310.0). Average length of stay in ED significantly increased by >1 hour (HWD1) and >2 hours (HWD2).

**Conclusions:**

Heatwaves significantly increase ED visits and workload even in a subtropical climate. The degree of impact is directly related to the extent of temperature increases and varies by socio-demographic characteristics of the patients. Heatwave action plans should be tailored according to the population needs and level of vulnerability. EDs should have plans to increase their surge capacity during heatwaves.

## Introduction

The effects of heatwaves and hot temperatures on mortality [1-7] and morbidity [8-11] are increasingly recognised. However, the type and magnitude of the effects vary from one place to another, depending on many factors including the climate, socio-demographic characteristics, community preparedness, and resilience of the population.

Previous studies show that high temperature and heatwaves are associated with higher mortality and years of life lost [12-15], as well as with increased risks of ambulance call-outs [16] and hospital admissions [14,17,18] due to heat-exacerbated health conditions such as cardiovascular disease (CVD), renal disease, diabetes, and non-external causes in general. However, the more direct impact of heatwaves on heat-related conditions such as heatstroke, heat exhaustion, and dehydration, classified under external causes of diseases by the International Classification of Diseases (ICD), is less studied [8,19,20], and is often embedded and analysed as part of the ‘other’ or ‘external’ causes of diseases, which also include conditions such as accidents and injuries, poisoning, and exposure to other forces of nature.

These heat-related conditions are often poorly recognised and ill-defined but capture those patients who may be physiologically distressed as a result of the effects of heat but do not fit into other diagnoses. Heat-related conditions can lead to severe consequences, sometimes even deaths [21]. They not only affect the health of the people, but also increase the burden on the health systems including the emergency health services [7,22]. It is therefore important to analyse the prevalence of heat-related conditions as a separate category in a region, rather than as a component of other diseases. Additionally, the monitoring of the presentation to the health care system of these conditions during heatwaves can provide timely surveillance information to improve decision-making, and target resources to where they are mostly required [23].

Public hospital emergency departments (EDs) are increasingly used by patients for a wide variety of conditions [24], and have been proven to be a useful surveillance tool to monitor the public health effects of heat events [25,26]. Despite a considerable amount of research on the effects of heat on mortality and health service utilisation in Brisbane [12-16,18], none have examined the effects of heat on daily ED presentations for conditions such as heatstroke and sun exposure. This study examines the association between heatwaves and the number of ED visits for heat illnesses further broken down by patients’ demographic characteristics, the acuity of their conditions and treatment outcomes.

## Materials and methods

### Study area

**Figure 1:**
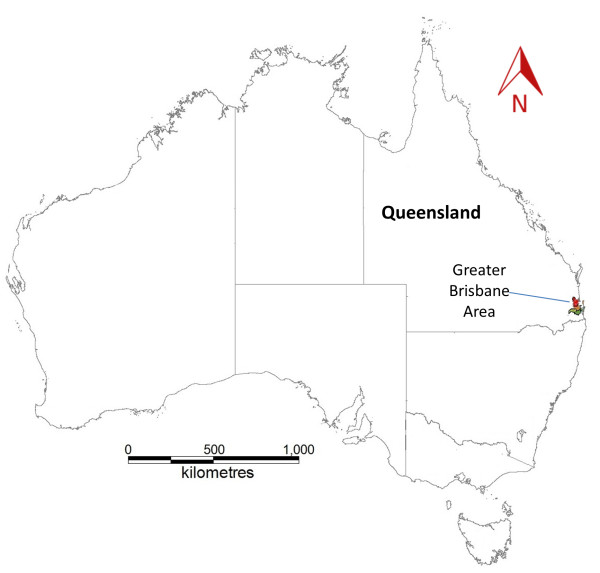
Map of the study area.

The study was conducted within the greater Brisbane area, a subtropical city located in South East Queensland, Australia. One ED was excluded due to incomplete data. The EDs were located in the Local Government Areas of Brisbane, Ipswich, Logan, Moreton Bay and Redland (Figure 1). In 2011-12, the greater Brisbane area had an estimated 2.2 million inhabitants, with an average annual growth rate of 2.4%, which has remained stable over the past decade [27].

### Heatwave definition

There is no official heatwave definition for Brisbane, and no universally accepted definition is available [18]. Therefore, following previous studies in this city [14,28,29], we adopted two measures, that is periods of at least two consecutive days having a daily maximum temperature ≥34°C (HWD1) or ≥37°C (HWD2). These thresholds closely match the 95^th^ and 99^th^ percentiles of maximum temperature (Tmax) in Brisbane for the study period.

### Study period and data

ED data for fiscal years of 2000 to 2012 for public hospital EDs in the greater Brisbane area were obtained from Queensland Health, which included 12 hospitals. One hospital was excluded as it only had ED data for 2007 to 2012; thus 11 EDs were included in this study.

The health data included de-identified records of daily presentations containing ICD codes, age, gender, triage category, dates and times of arrival and departure, and departure status. ICD codes for heat-related illnesses included: effects of heat and light such as heatstroke, sunstroke, heat syncope and heat exhaustion (ICD 10 code T67; ICD 9 code 992), and exposure to excessive natural heat (ICD 10 code X30; ICD 9 code E900.0). Since there were only eight cases with exposure to excessive natural heat, we combined these with the heatstroke group. We did not include dehydration because the dataset did not contain specific codes for dehydration. As previous studies show the heat effects vary across age groups [14,15,30], we re-coded age into four groups of 0 to 14 years, 15 to 64 years, 65 to 74 years, and 75+ years. ED patients are triaged at arrival for acuity of their conditions based on the Australian triage scale from 1 to 5, with 1 requiring immediate attention and 5 requiring care within 120 minutes [31]. For analysis purposes, we re-coded triage into three groups: 1 to 2 (high acuity), 3 (moderate), and 4 to 5 (low acuity). Departure status was provided as admitted or discharged. Length of stay in ED was calculated based on the time lapse between arrival and departure.

Daily Tmax and 24-hour average relative humidity data were obtained from the Bureau of Meteorology. Air pollution data including 24-hour average daily amounts of ozone (O_3_), particulate matter with <10 micrometers in aerodynamic diameter (PM_10_) and nitrogen dioxide (NO_2_) were provided by the Department of Science, Information Technology, Innovation and the Arts.

### Statistical analysis

The days were coded based on their Tmax and heatwave definition into three categories: non-hot day, single hot day, and heatwave day. We first assessed the difference in daily average number of ED presentations across these categories during the study period 2000 to 2012. We then recoded days as heatwave or non-heatwave, and used a poisson generalised additive model to examine the effect of heatwaves on ED visits after adjusting for potential confounders. We applied the generalised additive model as follows:

$$\mathrm{Ln}(E\left(Y_{t}\right)=\alpha +\sum_{i=1}^{p}\left(\beta_{i}x_{i}\right)+\mathrm{ns}\left(m,4\right)+\mathrm{ns}\left(y,\;4\right)+\sum_{j=1}^{q}s\left(x_{j},4\right)$$

Where *t* refers to the day of the observation; (*Y*_*t*_) denotes the observed disease counts on day *t*; *x*_*j*_ denotes the smooth function of relative humidity, O_3_, PM_10_ and NO_2_; *m* and *y* are the month and year, and natural cubic splines with 4 degrees of freedom were used to adjust for seasonal and long-term effects; *x*_*i*_ denotes the characteristic factors of the day of the week and heatwaves; *α* is the intercept term; *β* is the coefficient.

We then calculated the relative risks (RR) and 95% CI of attending on heatwave days versus non-heatwave days (non-hot and single hot days combined) by demographic and visit characteristics (acuity and discharge status). Analysis was restricted to summer months (December, January and February) to avoid the confounding effects of seasonality. To describe the RRs in the text, we have rounded the figures to one decimal point (rounded down if the second decimal digit was ≤5; rounded up if it was >5). The RR is reported as the number of times the risk increases. For example, RR = 4.98 is reported as 5 times. All analyses were performed in SPSS Statistics 21.0 (IBM Software, New York, USA) and R3.0.1 (R Foundation, Vienna, Austria).

Ethical approvals were granted by Queensland University of Technology Human Research Ethics Committee and Queensland Health Research Ethics Committee. As the data were provided in de-identified form, both committees waived the need for patient consent.

## Results

Table 1 shows low to moderate correlation between each pair of the environmental variables. Relative humidity was inversely associated with Tmax, O_3_ and PM_10_. NO_2_ was weakly but positively associated with PM_10_.

**Table 1:** Spearman’s correlation between environmental variables, December to February 2000 to 2012

	**Tmax**	**Relative humidity**	**O**_**3**_	**PM**_**10**_
**Relative humidity**	-0.35^**^			
**O**_**3**_	0.26^**^	-0.31^**^		
**PM**_**10**_	0.33^**^	-0.29^**^	0.36^**^	
**NO**_**2**_	-0.02	0.04	-0.03	0.13^**^

**P <0.01 (two-tailed). Number of days: 1,083. Tmax, daily maximum temperature in centigrade; O_3_, ozone; PM_10_, particulate matter with <10 micrometers in aerodynamic diameter; NO_2_, nitrogen dioxide.

Tmax ranged between 20.3°C and 40.2°C during the study period (Table 2) with a mean of 29.8°C (95% CI: 29.6°C, 29.9°C). Relative humidity ranged between 20.8% and 95.8% with a mean of 59.9% (95% CI: 59.2%, 60.6%). The daily mean was 0.02 ppm (95% CI: 0.02, 0.02) for O_3_, 18.4 μg/m^3^ (95% CI: 18.0, 18.8) for PM_10_, and <0.01 ppm (95% CI: 0.00, 0.00) for NO_2_.

**Table 2:** Descriptive characteristics of environmental variables, December to February 2000 to 2012

	**Tmax (°C)**	**Relative humidity (%)**	**O**_**3 **_**(ppm)**	**PM**_**10 **_**(μg/m**^**3**^**)**	**NO**_**2 **_**(ppm)**
**Mean**	29.83	59.90	0.02	18.43	<0.01
(95% CI)	(29.68, 29.97)	(59.21, 60.59)	(0.02, 0.02)	(18.03, 18.83)	(0.00, 0.00)
**SD**	2.43	11.90	0.01	6.71	0.00
**Minimum**	20.32	20.83	0.00	4.70	0.00
**Maximim**	40.20	95.83	0.04	81.31	0.01
**Median**	29.72	58.20	0.01	17.57	0.00
**95 pct**	33.92	82.44	0.03	29.74	0.01
**99 pct**	36.47	89.41	0.03	44.30	0.01

Tmax, daily maximum temperature in centigrade; O_3_, ozone; PM_10_, particulate matter with <10 micrometers in aerodynamic diameter; NO_2_, nitrogen dioxide; 95 pct, 95th percentile; 99 pct, 99th percentile.

**Figure 2:**
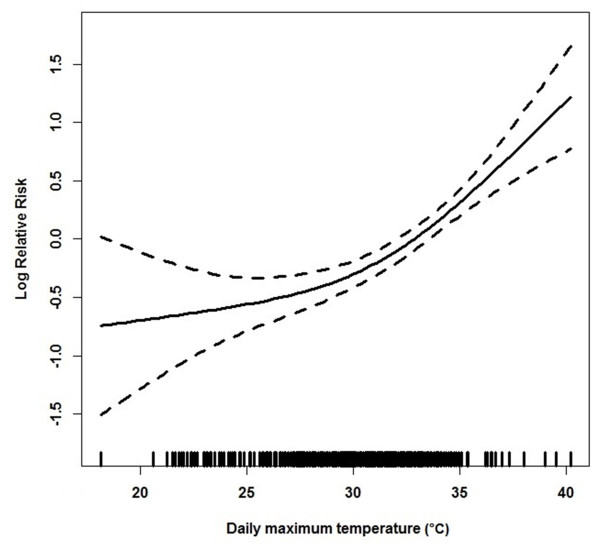
The association between daily maximum temperature in centigrade (Tmax) and emergency department (ED) presentations, December to February 2000 to 2012.

Of the 1,151 ED presentations for heat illnesses between 2000 and 2012, 628 cases (54.6%) occurred during summer periods. Figure 2 shows that the relative risk of heat-related illness visits increased with increasing temperatures. There appeared to be a more or less linear relation between ED presentations and Tmax between 30°C and 36°C, after which it became supra-linear.

Table 3 shows the number of presentations on non-hot, single hot and heatwave days. There were 19 single hot and 30 heatwave days from 2000 to 2012 in December to February based on HWD1, and two single hot and five heatwave days based on HWD2. The average of Tmax reached 35.5°C and 38.8°C during the heatwave days according to HWD1 and HWD2, respectively. Compared to non-heatwave days, the relative risks of heat-related illness visits on heatwave days increased 5.0 times (95% CI: 3.9, 6.4) and 18.5 times (95% CI: 12.0, 28.5) based on HWD1 and HWD2, respectively.

**Table 3:** ED presentations by non-hot, single hot and heatwave days, December to February 2000 to 2012

	**HWD1**			**HWD2**			
	**Non-hot**	**Single hot**	**Heatwave**	**Non-hot**	**Single hot**	**Heatwave**	**Total**
**Number of days**	1,034	19	30	1,076	2	5	1,083
**Presentations**	479	34	115	556	9	63	628
**Average/day**	0.46	1.79	3.83	0.52	4.50	12.60	0.58
**(95% CI)**	(0.41, 0.52)	(0.95, 2.63)	(1.47, 6.20)	(0.48, 0.56)	(3.52, 5.48)	(1.06, 24.14)	(0.49, 0.67)
RR* (95% CI)	**4.98 (3.88, 6.38)**			**18.53 (12.05, 28.49)**			

*Relative risk, heatwave days versus non-heatwave days (non-hot + single hot), controlled for relative humidity, particulate matter with <10 micrometers in aerodynamic diameter, ozone, nitrogen dioxide, day of week and long-term trends.

Table 4 shows the mean daily presentations and relative risks by patients’ demographic characteristics. Based on HWD1, females and males had 4.5 (95% CI: 3.1, 6.6) and 5.3 (95% CI: 3.8, 7.4) times more risk of ED visits with heat illness on heatwave versus non-heatwave days, respectively. However, based on HWD2, the relative risks increased 17.8 (95% CI: 9.8, 32.4) and 19.6 times (95% CI: 11.2, 34.4) for females and males, respectively.

**Table 4:** Mean daily emergency department presentations and relative risks by demographic characteristics, December to February 2000 to 2012

	**HWD1**				**HWD2**			
	**Non-hot**	**Single hot**	**Heatwave**	RR*	**Non-hot**	**Single hot**	**Heatwave**	RR*
**Gender**								
Female	0.19	0.89	1.60	4.55	0.21	4.00	5.60	17.79
(95% CI)	(0.16, 0.22)	(0.30, 1.49)	(0.40, 2.80)	(3.13, 6.61)	(0.18, 0.24)	(2.04, 5.96)	(−0.55, 11.75)	(9.76, 32.43)
Male	0.27	0.89	2.23	5.32	0.31	0.50	7.00	19.65
(95% CI)	(0.23, 0.31)	(0.18, 1.61)	(0.97, 3.49)	(3.83, 7.38)	(0.27, 0.35)	(−0.48, 1.48)	(1.06, 12.94)	(11.22, 34.42)
**Age group, years**								
0 to 14	0.06	0.11	0.2	3.00	0.06	0.50	0.80	29.85
(95% CI)	(0.04, 0.08)	(−0.03, 0.25)	(0.03, 0.37)	(1.19, 7.56)	(0.05, 0.07)	(−0.48, 1.48)	(0.07, 1.53)	(7.27, 122.56)
15 to 64	0.29	1.26	1.63	3.64	0.32	3.50	4.00	7.49
(95% CI)	(0.25, 0.33)	(0.58, 1.94)	(0.87, 2.39)	(2.56, 5.18)	(0.28, 0.36)	(2.52, 4.48)	(0.78, 7.22)	(3.93, 14.25)
65 to 74	0.05	0.26	0.67	7.29	0.06	0	2.20	23.54
(95% CI)	(0.04, 0.06)	(0.01, 0.51)	(0.25, 1.09)	(3.93, 13.53)	(0.04, 0.08)	--	(0.63, 3.77)	(8.99, 61.69)
75+	0.07	0.16	1.33	9.17	0.08	0.50	5.60	37.55
(95% CI)	(0.05, 0.09)	(−0.01, 0.33)	(0.01, 2.65)	(5.45, 15.44)	(0.06, 0.1)	(−0.48, 1.48)	(−1.67, 12.87)	(18.34, 76.86)
**Indigenous status**								
Indigenous	0.01	0	0.03	1.63	0.01	0	0	0
(95% CI)	(0, 0.02)	--	(−0.04, 0.1)	(0.14, 19.13)	(0, 0.02)	--	--	--
Non-indigenous	0.44	1.79	3.77	4.91	0.50	4.50	12.40	18.28
(95% CI)	(0.39, 0.49)	(0.95, 2.63)	(1.4, 6.14)	(3.82, 6.31)	(0.44, 0.56)	(3.52, 5.48)	(0.71, 24.09)	(11.85, 28.22)

*Relative risk, heatwave days versus non-heatwave days (non-hot + single hot), controlled for relative humidity, particulate matter with <10 micrometers in aerodynamic diameter, ozone, nitrogen dioxide, day of week and long-term trends. -- not applicable.

Regarding age, based on HWD1, the relative risks of presentations with heat illness on heatwave days increased 3.0 times (95% CI: 1.2, 7.6) for ages 0 to 14 years, 3.6 times (95% CI: 2.6, 5.2) for ages 15 to 64 years, 7.3 times (95% CI: 3.9, 13.5) for ages 65 to 74 years, and 9.2 times (95% CI: 5.4, 15.4) for ages 75 years and above. On the other hand, based on HWD2, the relative risks increased 29.8 times (95% CI: 7.3, 122.6), 7.5 times (95% CI: 3.9, 14.2), 23.5 times (95% CI: 9.0, 61.7) and 37.5 times (95% CI: 18.3, 76.9) for ages 0 to 14 years, 15 to 64 years, 65 to 74 years and 75+ years, respectively.

Considering the indigenous status, only 11 (1.7%) patients were identified as indigenous. Based on HWD1, the increases were not statistically significant, and based on HWD2, all indigenous patients attended with heat illness on non-hot days. For the non-indigenous patients, the relative risks increased 4.9 (95% CI: 3.8, 6.3) times according to HWD1, and 18.3 times (95% CI: 11.8, 28.2) based on HWD2.

Table 5 shows average daily presentations and relative risks by illness characteristics. Based on HWD1, the relative risks of presentations with high, moderate and low acuity increased nearly five times on heatwave days. However, based on HWD2, the relative risks increased 81.7 times (95% CI: 21.5, 310.0) in the high acuity category, 17.2 times (95% CI: 9.4, 31.6) in the moderate category, and 9.9 times (95% CI: 5.2, 19.1) in the low acuity group. Regarding the departure status, based on HWD1, the relative risks of admission or discharge increased almost equally at close to five times. However, based on HWD2, the relative risk of admission with heat illness increased 31.3 times (95% CI: 15.2, 64.4) and the risk of discharges increased 14.7 times (95% CI: 8.2, 26.2). The average length of stay in the ED with heat illness also increased significantly on heatwave versus non-heatwave days by over one hour based on HWD1, and over two hours based on HWD2.

**Table 5:** Average daily emergency department presentations and relative risks by illness characteristics, December to February 2000 to 2012

	**Heatwave day ≥34°C**				**Heatwave day ≥37°C**			
	**Non-hot**	**Single hot**	**Heatwave**	RR*	**Non-hot**	**Single hot**	**Heatwave**	RR*
**Acuity (triage)**								
High (1 to 2)	0.04	0.16	0.63	4.69	0.04	0.50	3.80	81.72
95% CI	(0.03, 0.06)	(−0.01, 0.33)	(−0.23, 1.49)	(2.30, 9.57)	(0.03, 0.06)	(−0.48, 1.48)	(−0.73, 8.33)	(21.54, 310.01)
Moderate (3)	0.28	0.89	1.83	4.89	0.31	1.50	5.20	17.21
95% CI	(0.24, 0.32)	(0.26, 1.53)	(0.9, 2.77)	(3.47, 6.89)	(0.27, 0.35)	(−1.44, 4.44)	(1.21, 9.19)	(9.37, 31.62)
Low (4 to 5)	0.14	0.74	1.37	4.84	0.16	2.50	3.60	9.95
95% CI	(0.11, 0.16)	(0.27, 1.21)	(0.62, 2.12)	(3.16, 7.42)	(0.13, 0.19)	(−0.44, 5.44)	(0.01, 7.19)	(5.19, 19.08)
**Departure status**								
Admitted	0.09	0.42	1.33	5.16	0.10	2.00	5.80	31.31
95% CI	(0.07, 0.11)	(−0.01, 0.85)	(−0.04, 2.71)	(3.24, 8.21)	(0.08, 0.12)	(−1.92, 5.92)	(−1.72, 13.32)	(15.23, 64.37)
Discharged	0.34	1.26	2.03	4.87	0.38	2.50	4.80	14.69
95% CI	(0.3, 0.39)	(0.5, 2.03)	(0.82, 3.24)	(3.51, 6.77)	(0.33, 0.43)	(−2.4, 7.4)	(−1.09, 10.69)	(8.22, 26.24)
**Length of stay (hh:mm)**				***F*-test ( *P *)**				***F*-test ( *P *)**
Mean	3:47	3:20	4:45	5.27	3:45	2:26	5:53	15.60
Minimum	0:08	0:44	0:21	(0.005)	0:08	0:44	1:18	(0.000)
Maximum	24:42	8:34	20:55		24:42	4:17	20:54	
SD	2:54	1:43	3:53		2:48	0:59	4:33	

*Relative risk, heatwave days versus non-heatwave days (non-hot + single hot), controlled for relative humidity, particulate matter with <10 micrometers in aerodynamic diameter, ozone, nitrogen dioxide, day of week and long-term trends.

## Discussion

This is the first study oF heat illness and ED visits associated with heatwaves in Brisbane, and complements previous work on the effects of heat on mortality, hospital admissions and ambulance utilisation in this subtropical climate [13-16]. Our results show that as the temperature increased so did the likelihood by all gender and age groups of attending EDs with heat-related illness. It also confirms that the magnitude of impact can greatly vary by how a heatwave is defined [18]. For instance, based on HWD1, the relative risks increased three times in the 0 to 14 years age group, whereas based on HWD2, the impact elevated by 29 times. Similarly in the oldest age group (age 75 years and older), the RR increased from 9 to 37 times according to HWD1 and HWD2, respectively. Although the impact by triage category (acuity) and departure status were similar based on HWD1, the risk of attending with a higher acuity condition based on HWD2 was much higher than the risk of attending with a lower acuity condition. Furthermore, the chance of admission was double the chance of discharge on HWD2, and patients were considerably more likely to stay longer in the ED.

Consistent with the limited number of studies in this area, our findings confirm the health impact of heatwaves. Mayner *et al*. [32] showed that during the two heatwave periods in Adelaide, South Australia, in 2009, the number of heat-related presentations to one ED increased 21 and 17 times versus comparable pre-heatwave periods. Another study in Sydney, Australia, also found that ED visits with heat-related illnesses increased over seven times during the 2011 heatwave compared to the average number of such visits in previous years, with 100% excess rate per 100,000 population amongst people aged 75+ [33]. Other Australian studies show that hospital admissions for heat-related conditions increased over three times in Sydney [19] and nearly six times in New South Wales [8] during extreme heat periods. Although both of these figures are close to the ED admissions in our study based on HWD1, they are far lower than the HWD2 figure (31.3 times). One reason may be that these studies also included volume depletion as a proxy for dehydration in their analyses. As volume depletion could happen on non-hot days as a result of other factors besides heat exposure (for example, strenuous activities), the inclusion may have affected the RR estimates. Furthermore, the differences show the extent to which the heat effects depend on the location and measurement standards.

During the 2006 California heatwave, heat-related ED visits increased 6.3 times (95% CI: 5.6, 7.0) and admissions 10.1 times (95% CI: 7.7, 13.4) [34]. The same study reported that the age-specific ED visits increased 6.1, 5.4 and 10.8 times for age groups 0 to 4, 5 to 64 and 65+ years, respectively. Again, although these figures are close to HWD1 results, they are considerably lower than the HWD2 findings. In addition to differences in heatwave definition, the difference may be attributed to design differences, as the California study compared one heatwave period with a reference period using a descriptive approach, whereas our study used a time-series study design. Furthermore, population characteristics and community preparedness may explain some of the differences. The age groups were not directly comparable as the two studies used different age brackets, although both reported similar patterns.

Our study also showed that the heat effects varied across demographic characteristics. There are various possible explanations. Children may be more vulnerable due to lack of proper care and adaptation behaviour [30], whereas older patients may be more at risk due to a ‘less developed thermoregulation or their low self-care ability’, which may be compounded by health comorbidities [35]. Other factors, such as socio-economic status, living conditions, social support, working environment (for example, working outdoors during the day), and access to preventative and protective measures, may at least partly explain and contribute to the degree of exposure to risk and the effects of heatwaves [21,36-38].

Our data did not show any association between indigenous status and heat-related ED visits on hot or heatwave days based on HWD2. This is counter-intuitive as other studies show that indigenous communities are higher users of ED services than the non-indigenous [39], and are assessed to be highly vulnerable to climate change impacts due to chronic health conditions and lower socio-economic status [40]. There is no certain explanation for this finding. One speculation may be that due to higher prevalence of other diseases such as diabetes, cardiovascular and renal problems among the indigenous than non-indigenous groups [41], the presentation was not coded as a heat-related illness, but as the underlying health condition (for example, renal problems) exacerbated by heat. It may also be hypothesized that the indigenous people are better adapted to heat. Further research is required to provide a conclusive response.

We also found that risks of high-acuity visits and admissions with heat illness increased disproportionately during extreme heatwaves (HWD2 compared to HWD1). This may be attributed to lack of preparedness, at least in some sections of the community, for extremely hot periods. Brisbane has lengthy periods of warm temperatures in summer, and most residents may be acclimatised and prepared for normally hot days. However, with changing climate, population aging and high rate of immigration in this region [27], such assumptions cannot be taken for granted. Currently, there is no research available to show how prepared different groups of people are in facing the warming climate in Queensland, including Brisbane. However, studies in other countries show that people who see themselves personally vulnerable are more likely to take action to protect against heat harm [42].

This study has major strengths. To our knowledge, this is the first study to examine the association of heatwaves with heat illnesses in a subtropical climate. As the health impacts of heatwaves are highly location-specific, this study contributes to understanding of the acute effects of heat in warmer locations. Second, although studies have shown that ED visits generally increase on heatwave days to some extent, our findings show that presentations with heat illness are disproportionately much higher than other illnesses. Finally, in addition to examining the heat illness visits, we analysed the impact of heatwaves on the acuity of the condition, admission status and length of stay. This enabled us to show a more accurate picture of how increasing temperatures can increase the health system workload and reduce bed capacity. On heatwave days, the average length of stay in the ED increased significantly, due potentially to an increased number of patients and higher severity of their conditions, as determined by higher RR of acuity and admission. These can have considerable implications for the emergency health system which is under constant pressure due to excess demand and overcrowding [24,43].

The study had certain limitations. First, due to the unavailability of data, the study population was limited to patients attending 11 public hospital EDs and who were diagnosed as having heatstroke or related conditions. Patients using private EDs, primary care services, or those who did not seek care but were affected, were not included. These, plus exclusion of one ED due to incomplete data, underestimate the true extent of heatwave effects in Brisbane. In particular it does not include those in whom the heat has exacerbated other chronic or acute illnesses. Second, because of small numbers, we were unable to establish whether the indigenous group was affected differently during heatwaves. Only 11 cases (1.7%) were identified as indigenous. More complete data and a larger sample are needed for such analyses. In addition, the non-indigenous group comprises patients from a wide range of other ethnic groups. Better and more detailed data are required to analyse ethnic differences based on these factors. Third, we could not obtain data such as patient’s location at the time of illness, arrival method, comorbidities and follow-up data after admission to inpatient wards or discharge. Such data would have been able to provide a more comprehensive picture of the impact of heatwaves on patients and the emergency health system. Last, we did not have information on individual exposure to heat. Also it is likely that heatstroke may have been due to other heat sources, particularly on non-hot days. Such information can only be obtained through direct monitoring of patients, which was outside the scope of this study.

## Conclusions

More patients and sicker patients (as determined by the increase in the average number and relative risk of presentation in high acuity categories) attend EDs during heatwaves and hot days, causing an extra workload for the emergency health system which is already under stress. Targeted protective measures are proven to assist with reducing these impacts [42]. ED data can be reliably used as a monitoring and surveillance tool for timely detection of the syndromes and early effects of heatwaves [26]. The data can be used to help the health service providers prepare for the potential effects, and to ensure that the limited resources are allocated to those who need them most.

## Key messages

•Even in a subtropical climate where people are better acclimatised to warmer temperatures, hot temperatures and heatwaves can still have serious acute health effects on the population.

•The magnitude of impact varies across age groups. Children, young teenagers and the elderly are particularly vulnerable.

•Heat health warning, protection and intervention plans and protocols should be developed to respond to heatwave impacts, advise the community of the health dangers, and implement strategies to minimise the risk.

•Health education messages and programmes should specifically target these high risk groups and their carers, such as parents of young children, daycarers, retirees and independent living elderly, clubs and other relevant agencies to inform them of the risks and how to protect themselves.

•Health service providers including the EDs should prepare for and increase their surge and admission capacities to deal with the increase in demand for such services as a result of high temperatures, particularly as climate change progresses.

## Abbreviations

ED: emergency department; HWD1: heatwave day ≥34°C; HWD2: heatwave day ≥37°C; ICD: International Classification of Diseases; NO2: nitrogen dioxide; O3: ozone; PM10: particulate matter with <10 micrometers in aerodynamic diameter; ppm: parts per million; Tmax: daily maximum temperature in centigrade.

## Competing interests

The authors declare that they have no competing interests.

## Authors’ contributions

GT and ST conceived the study and participated in the design. GT, PA and GF contributed to the acquisition of data. GT and WY analysed and interpreted the data and prepared the first draft. GT, ST, PA and GF interpreted the results and revised the drafts. All authors critically reviewed the drafts for intellectual content. All authors have given approval of the final manuscript. All authors agree to be accountable for all aspects of the work in ensuring that questions related to the accuracy or integrity of any part of the work are appropriately investigated and resolved.
